# Normalography: A Novel Imaging Technique for Visualizing Pixel-Wise Surface Normal Distributions

**DOI:** 10.3390/s26154725

**Published:** 2026-07-25

**Authors:** Shinichi Inoue, Yoshinori Igarashi, Seiji Suzuki

**Affiliations:** 1 Faculty of Engineering, Tokyo Polytechnic University, 5-45-1 Iiyamaminami, Atsugi 243-0297, Kanagawa, Japan; 2 CHUO PRECISION INDUSTRIAL CO., LTD., 65 Shirasaka Miwadai, Shirakawashi 961-0835, Fukushima, Japan; igarashi@chuo.co.jp (Y.I.);

**Keywords:** normalography, surface normal measurement, optical sensing, surface inspection, machine vision, multispectral imaging, reflectance measurement, multi-angle illumination

## Abstract

**Highlights:**

**What are the main findings?**

- We propose a novel imaging technique, termed normalography, for visualizing pixel-wise surface normal distributions.
- The technique combines a multi-angle collimator with multispectral illumination to simultaneously acquire surface normal information from multiple directions.

**What are the implications of the main findings?**

- An imaging system was developed to capture surface normal directions across a 1024 × 1024-pixel field of view using multispectral illumination and simultaneous multi-angle reflectance measurements.
- The proposed normalography enables real-time surface inspection and online quality monitoring in industrial manufacturing processes.

**Abstract:**

Surface quality is a critical indicator of product performance, creating a growing demand for real-time surface inspection in industrial manufacturing. However, conventional surface normal measurement techniques require sequential measurements with varying illumination or observation angles, making them unsuitable for high-speed online inspection. To overcome this limitation, this paper proposes a novel imaging technique, termed normalography, for visualizing pixel-wise surface normal distributions. Analogous to thermography, normalography visualizes the spatial distribution of surface normal directions over a material surface. The proposed method targets highly glossy and smooth surfaces and is based on reflectance measurements. A multi-angle collimator was developed to simultaneously illuminate the target surface from multiple incident directions, while multispectral illumination was employed to distinguish the reflected light corresponding to each direction. An imaging system incorporating red, green, and blue illumination sources enables single-shot acquisition of surface normal information over a 1024 × 1024-pixel field of view. The proposed normalography enables camera-like real-time visualization of surface normal distributions without sequential image acquisition, demonstrating its potential for online surface inspection and quality monitoring in industrial manufacturing.

## 1. Introduction

In industrial sectors, the evaluation of material surface properties is a critical process that strongly influences product performance and reliability. In particular, measurements of height distribution and surface roughness associated with surface topography are widely conducted. On the other hand, when materials are used as final products, surface properties directly affect the appearance perceived by human users [1,2,3,4,5,6]. Fine surface asperities modify visual impressions such as gloss through their influence on the reflection and scattering of light. A macroscopic surface can be regarded as an assembly of mesoscopic facets, each characterized by a surface normal defined as the vector perpendicular to the local surface. Variations in these surface normals reflect surface roughness, produce irregular reflection characteristics, and strongly influence the perceived texture of the material. Although the appearance of a material can be recorded using photographs, such records represent only a specific phenomenon under a particular illumination condition. Consequently, there is a growing need for methodologies that can quantitatively capture these phenomena from a physical perspective. The surface properties of materials are often continuously developed during the production process, and there is a high demand for sensing technologies that enable online inspection. This paper presents a method for measuring the spatial distribution of surface normal directions with high speed. By integrating surface normal sensing with imaging technology, the proposed method enables rapid visualization of material surface characteristics over a wide field of view.

**Figure 1:**
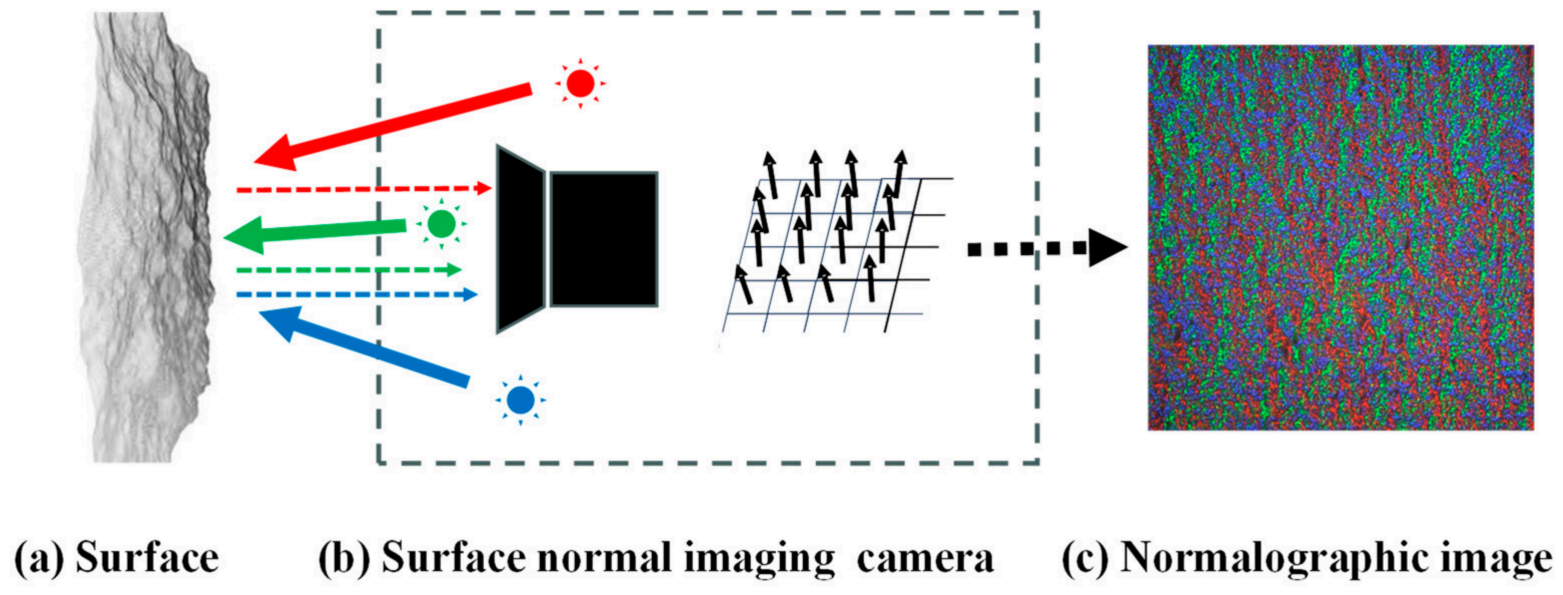
Concept of normalography. (**a**) Measured surface; (**b**) surface normal imaging camera developed in this study; (**c**) example of a normalographic image visualizing pixel-wise surface normal distributions using color coding.

We propose a novel imaging methodology, termed normalography, for visualizing the spatial distribution of surface normal directions (Figure 1). Analogous to thermography [7], which visualizes temperature distributions, normalography generates an image representation of pixel-wise surface normal distributions over a material surface. This visualizes pixel-wise surface normal directions rather than the three-dimensional height of the sample surface.

Visual evaluation of reflective properties is still widely performed, particularly in fields where appearance plays a critical role [3,4,5,6]. Motivated by this practice, we propose measuring the high-resolution distribution of surface normals based on reflective properties.

**Figure 2:**
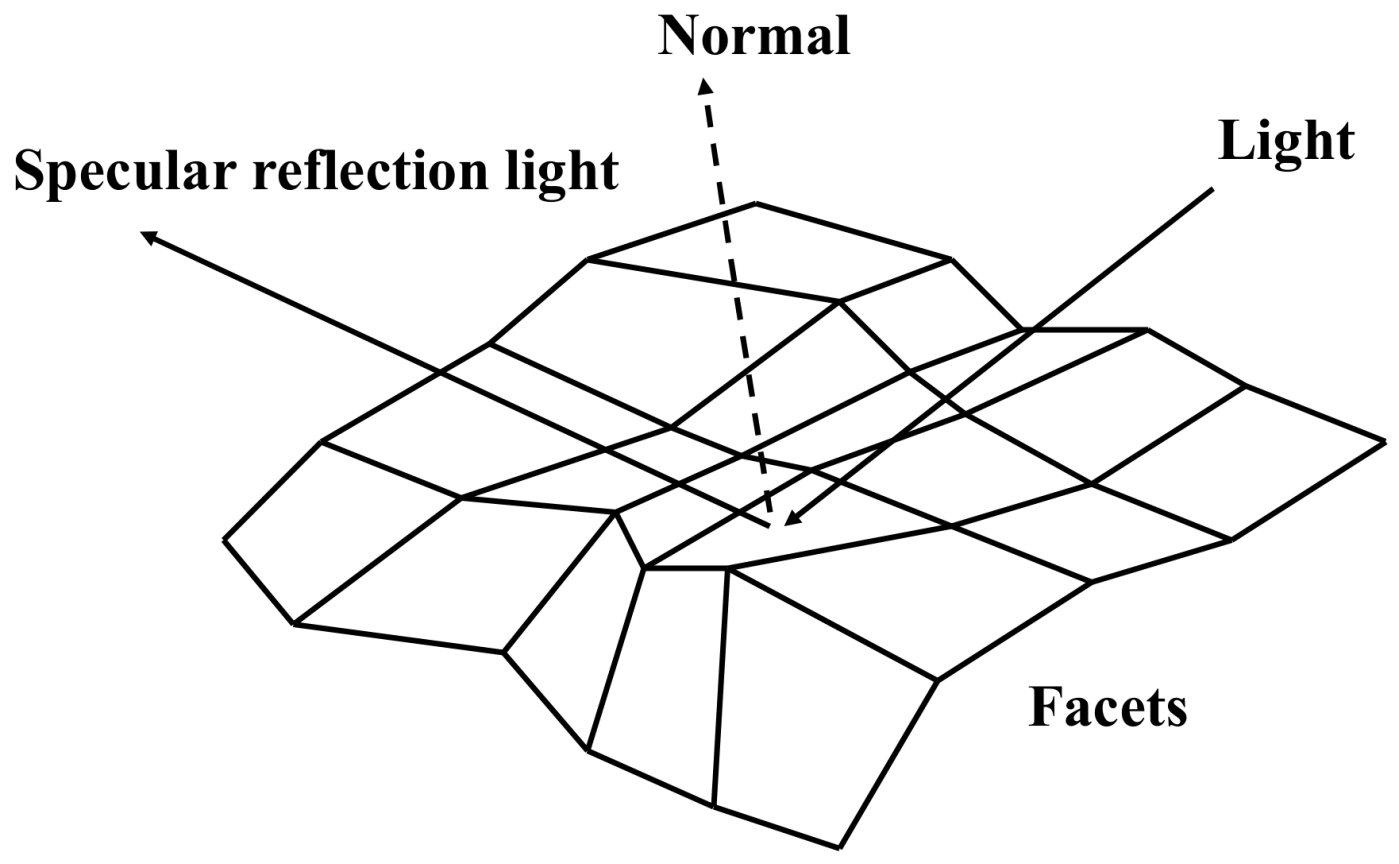
Schematic illustration of a macroscopic surface composed of mesoscopic facets. The direction of specular reflection varies according to the orientation of each surface normal.

**Figure 3:**
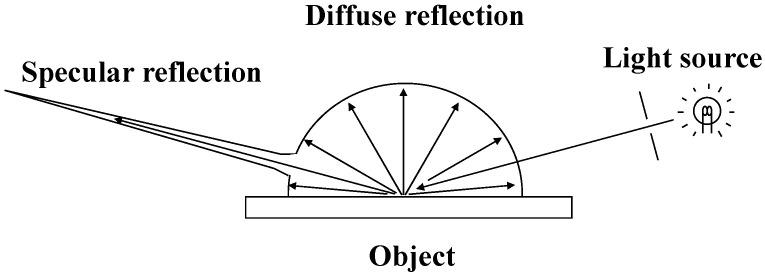
Schematic illustration of the dichromatic reflection model. The BRDF describes the angular distribution of reflected light as a combination of diffuse and specular reflection components.

The distribution of surface facets and their normals is illustrated in Figure 2. In three-dimensional space, a surface normal is defined as a vector perpendicular to the corresponding local tangent facet. The reflected light distribution from each facet depends on its surface normal vector. The distribution of reflected light is illustrated in Figure 3. This distribution of reflectance intensity is described by the bidirectional reflectance distribution function (BRDF), which defines how light from a source is reflected by a surface [8]. In the dichromatic reflection model, the reflected light intensity is expressed as the sum of diffuse and specular components. As shown in Figure 3, part of the incident light is absorbed, scattered, and reflected in many directions; this is referred to as diffuse reflection. Printed images are primarily observed through diffuse reflection. Specular reflection, in contrast, is mirror-like and highly directional. The physical basis of gloss is specular reflection. Variations in surface normals lead to changes in specular reflection near the specular direction. The corresponding reflectance distribution typically exhibits a convex profile with a peak at this direction, where the reflected intensity is maximized.

The proposed system estimates the surface normal direction based on the known direction of incident light and the detected direction of specular reflection. Because specular reflection has a narrow angular spread and is much stronger in intensity than diffuse reflection, the surface normal direction can be detected easily and reliably.

Various methods based on deflectometry have been investigated as a means of directly measuring the spatial distribution of surface normal vectors [9,10,11,12]. Methods using photometric stereo and AI-based estimation have also been proposed to obtain depth maps [13,14,15]. Other approaches measure the spatial distribution of surface height and derive surface normals from its gradients. Stylus-type profilometers, white-light interferometers, and confocal microscopes are commonly used to measure high-resolution surface structures [16,17,18]. Methods for measuring the normal vectors of a single surface point using reflectance are well established. The reflectance of an object’s surface under varying incident light angles can be measured using a technique known as gonio-reflectance [19,20]. However, such measurements are generally limited to a single point rather than a spatial distribution, and they require scanning the surface using complex mechanisms and a considerable amount of measurement time [20,21]. Furthermore, a key difference is that conventional methods for measuring surface normal distributions did not allow for rapid measurements.

A previously reported method by the authors achieved high-resolution measurement of surface normal vectors with high angular accuracy [22]. However, each measurement required several minutes, limiting its applicability to online inspection. In this study, we have improved this method to enable real-time detection using a new solution. This method has prioritized detection speed over angular resolution. This trade-off enables practical deployment for industrial inspection and production monitoring, where immediate visualization is often more valuable than highly precise offline analysis. There has long been a need for methods to measure the spatial distribution of surface normals in real time. In this paper, we propose a method for visualizing the direction of the surface normal at each pixel location in a single shot. We have developed a prototype based on the proposed method and present the measurement results for several samples. We verify and report on the angles and ranges of the normals that can be measured using this prototype. We also discuss potential pathways for improving measurement range, angular resolution, and quantitative normal-vector estimation. In the current prototype, the surface normal direction is classified into eight angular categories for high-speed imaging. Future developments will focus on increasing the number of detectable normal directions and improving angular resolution.

Although the proposed system employs established optical concepts, its originality lies in the development of a multi-angle collimator illumination unit capable of generating multiple precisely controlled collimated illumination beams simultaneously over the full measurement area. This optical implementation eliminates sequential angular scanning and forms the key enabling technology for real-time normalography. Combined with multispectral encoding, it allows direct imaging of pixel-wise surface normal distributions immediately after image acquisition.

## 2. Methods

This section explains the proposed method from a theoretical perspective and presents the specifications of the apparatus implemented in practice. The apparatus specifications and experimental conditions are consistent with those used in the experiments. The multi-angle collimator is the core optical component of the proposed system. Unlike conventional collimated illumination, it simultaneously generates multiple parallel beams with accurately controlled incident angles while maintaining spatially uniform illumination across the measurement area.

### 2.1. Coordinate System and Notation

In this study, the target surface is regarded as an assembly of small planar facets, each characterized by a surface normal direction. The two-dimensional coordinates on the target surface are denoted by x and y, while the *z*-axis is defined as the direction perpendicular to the nominal surface. A right-handed Cartesian coordinate system is adopted throughout this paper.

**Figure 4:**
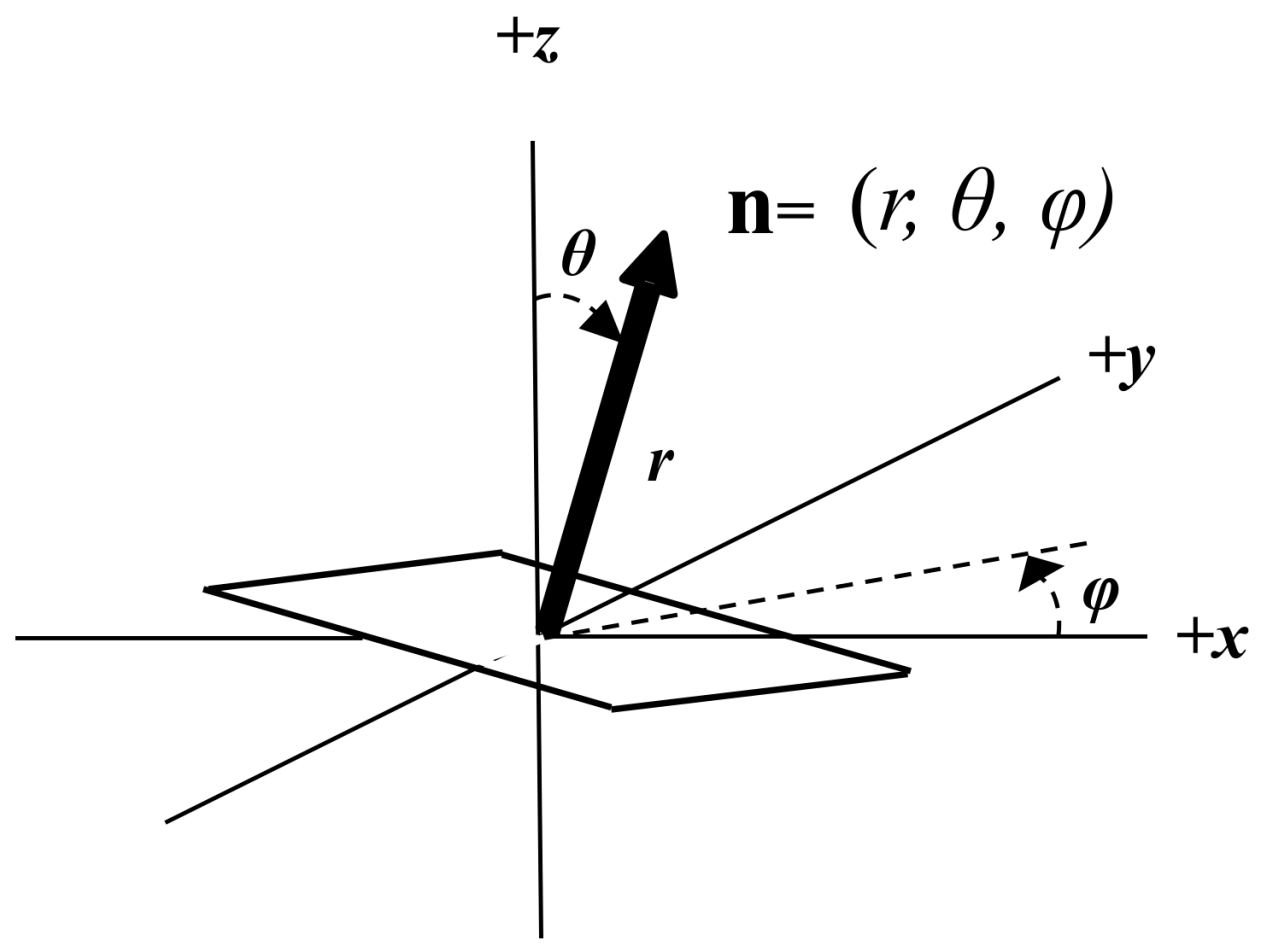
Schematic diagram illustrating the surface normal vectors. Here, **n** denotes the surface normal vector.

The surface normal direction is represented using a spherical coordinate system, as illustrated in Figure 4. In this coordinate system, the surface normal vector **n** is described by a radial distance *r*, a polar angle *θ*, and an azimuthal angle *φ*, and is expressed as

(1) n=(r,θ,φ),

where *r* = 1.0 for the unit surface normal vector used throughout this study.

Since only unit surface normal vectors are considered in this study, the radial distance is fixed at *r* = 1.0. Therefore, the surface normal direction is uniquely determined by the polar angle *θ* and azimuthal angle *φ*. The *z*-axis is defined as the zenith direction. For *θ* = 0.0°, the azimuth angle *φ* becomes arbitrary. 

### 2.2. Principle of One-Shot Surface Normal Imaging

The proposed method is based on specular reflectometry. The surface normal vector is estimated as follows. According to the law of specular reflection, the surface normal vector can be estimated as the half-vector **h** between the incident light vector **l** and the specular reflection vector **s** [23]. Therefore, the surface normal vector **n** is expressed as

(2) $$n=h=\frac{l+s}{\left|l+s\right|},$$

The proposed method simultaneously detects multiple surface normal directions over the entire imaging field. This method covers a target range of surface normal angles and visualizes the surface normal distribution at the pixel level all at once.

We apply multispectral imaging techniques, allowing us to separate light using color filters. Each incident illumination angle is assigned a specific spectral band, enabling us to detect specular reflected light with the same color spectrum, allowing direct separation of illumination directions using the RGB channels of a standard color camera.

In developing our surface normal imaging system, we are focusing on two key technologies: multi-angle illumination using collimators and limited-direction inspection imaging using telecentric optics.

### 2.3. System Implementation

The proposed method is based on reflectometry. The key innovation is a multi-angle collimator that simultaneously illuminates the target surface from multiple incident directions. This approach significantly reduces system complexity and enables high-speed real-time imaging. Achieving simultaneous multi-angle illumination with angular intervals below 1.0° is challenging using conventional illumination systems.

**Figure 5:**
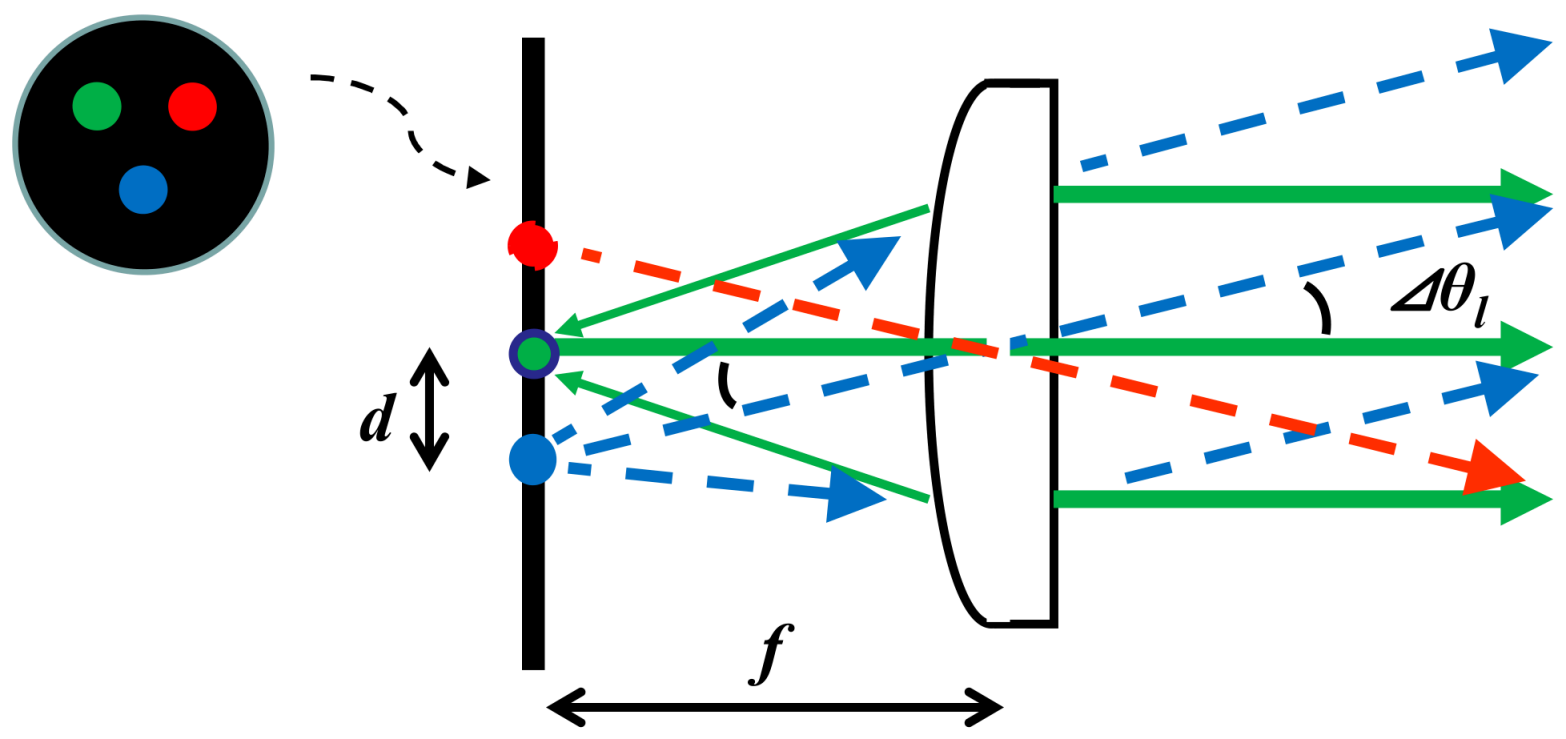
Principle of the multi-angle collimator. Multiple light sources are positioned at calculated offsets from the optical axis to generate collimated illumination at different incident angles.

A schematic diagram of the multi-angle collimator illumination system based on collimated lighting is shown in Figure 5. In collimator optics, a focal point exists on one side of a lens, while parallel light emerges on the other side. When a light source is placed at the focal point, the illumination system emits parallel (collimated) light.

According to the principles of geometrical optics, a collimator lens converts an off-axis point located on its focal plane into a parallel beam propagating at an angle Δ*θ_l_* with respect to the optical axis. Therefore, the radial displacement *d* on the focal plane is related to the propagation angle as follows:

(3) $$d=f\cdot tan(\Delta \theta_{l}),$$

where *f* is the focal length of the collimator lens [23].

In conventional configurations, the light source is positioned at the focal point on the optical axis. As shown in Figure 5, the newly developed device utilizes multiple light sources. By calculating the positions of the light sources, parallel light can be irradiated at the desired illumination angle. Because it is a collimator, the angle of incident light is spatially uniform across the entire sample surface.

**Figure 6:**
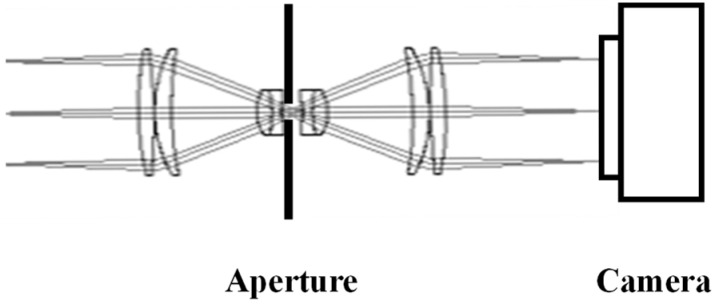
Principle of the telecentric imaging system. The observation direction is maintained uniformly across the entire field of view, enabling spatially consistent measurement of specular reflection.

A telecentric optical system selectively accepts light rays that are nearly parallel to the optical axis in Figure 6. The allowable angle of this passing light can be controlled by the aperture. Experimental results will be reported later. This ensures a uniform observation angle across the entire measurement surface. In the prototype, the observation vector is in the zenith direction, the *z*-axis.

### 2.4. Concept of Normalography

The proposed method combines multi-angle illumination, multispectral separation, and telecentric imaging to visualize the spatial distribution of surface normal directions in a single image acquisition. By detecting surface normal directions at each pixel location, the system generates a normalographic image representing the surface normal distribution over the target surface. The developed apparatus functions as a surface normal imaging camera capable of real-time measurement. In the following sections, the prototype system and its measurement performance are presented, followed by a discussion of potential extensions and scalability of the proposed method.

## 3. Experiment and Results

A prototype apparatus based on the proposed method was developed and evaluated. The system measures surface normal directions over a field of 1024 × 1024 sampling points with a spatial sampling interval of 0.02 × 0.02 mm per pixel. In the current implementation, the normal direction is classified into eight categories: a reference flat direction, six surrounding directions distributed at azimuthal intervals of 60° with a polar angle of approximately 0.5°, and an additional category representing directions outside the predefined angular range. This category may correspond to larger surface inclinations, scratches, contamination, or other surface anomalies. Using the developed apparatus, normalographic images were acquired for various materials, including plastic, ceramic tile, inkjet paper, and aluminum sheets.

### 3.1. Developed Surface Normal Imaging Camera

**Figure 7:**
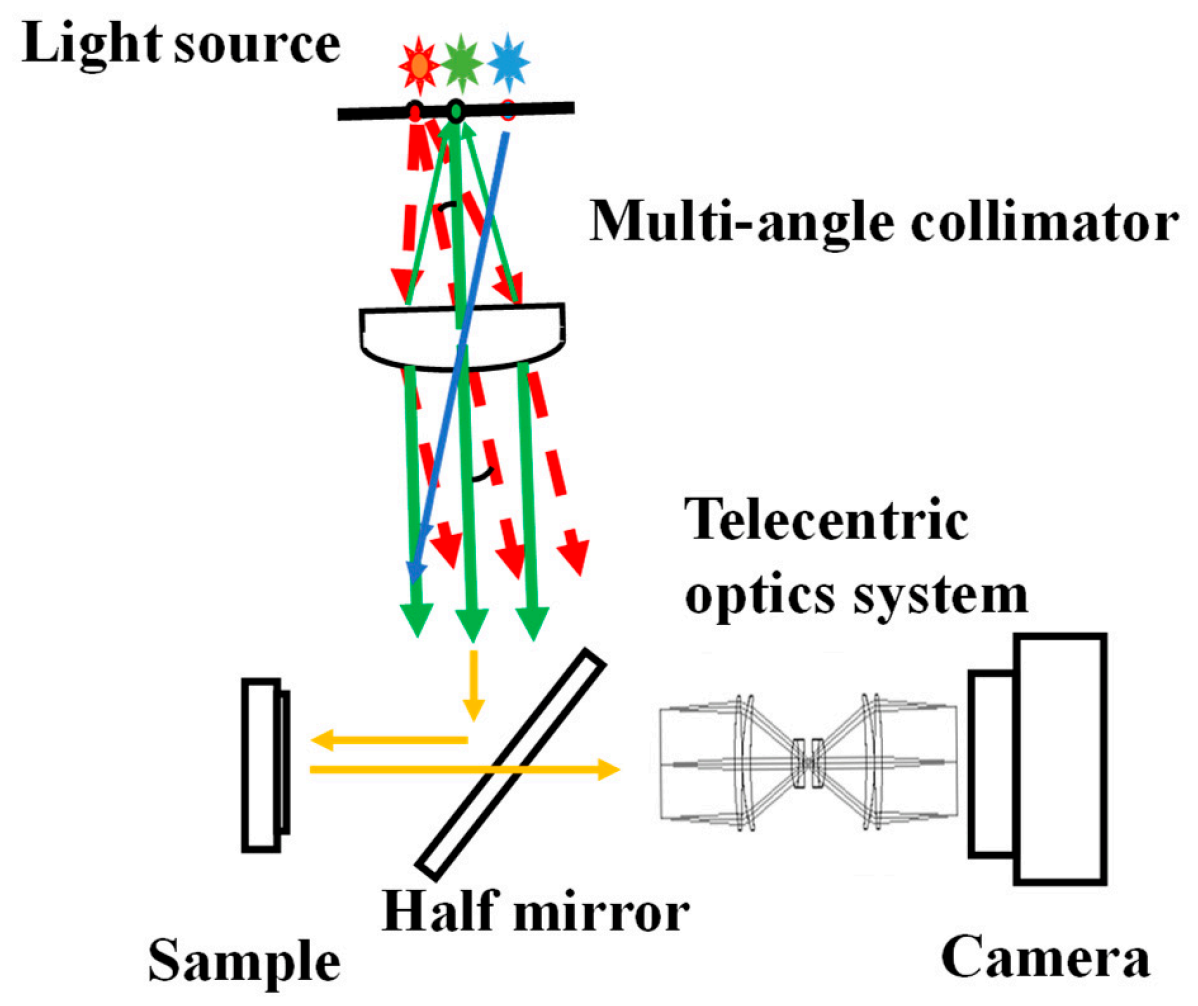
Schematic diagram of the developed surface normal imaging camera. The system consists of a multi-angle collimator illumination unit, a half mirror, telecentric imaging optics, and a CMOS camera.

The developed measurement apparatus is shown in Figure 7. Collimated light beams generated by the multi-angle collimator are reflected by a beam splitter and directed onto the sample surface. Specular reflected light is captured by a CMOS camera equipped with telecentric optics. The imaging sensor is a DFK 33UX249 CMOS camera (The Imaging Source, Bremen, Germany). The telecentric lens is a 0.30× SilverTL Telecentric Lens (Edmund Optics, Barrington, NJ, USA) with a diameter of 46 mm, an object-side numerical aperture of 0.025, and an f-number of 22. The observation direction is fixed along the surface normal (0°). The effective measurement area is 20.48 × 20.48 mm, corresponding to an image size of 1024 × 1024 pixels. All measurements were performed in a darkroom environment with the sample positioned on a precision stage. The prototype apparatus was developed by CHUO PRECISION INDUSTRIAL CO., LTD. (Tokyo, Japan).

### 3.2. Prototype Multi-Angle Collimator Illumination Unit

The multi-angle collimator was designed using a lens system with a diameter of 46 mm and a focal length of 150.0 mm. An LED light source with a 0.8 mm aperture was placed at the focal point to generate collimated illumination.

**Figure 8:**
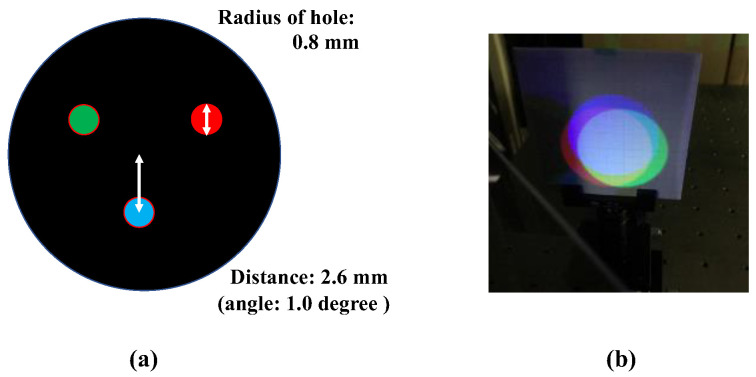
(**a**) Schematic diagram of the aperture mask used for multi-angle collimator. (**b**) Photograph of the experimental setup showing overlapping red, green, and blue illumination regions defining the measurement area.

The system employs multiple illumination channels, each equipped with an optical bandpass filter. In this experiment, a mask with three apertures was positioned at the focal plane of the collimator, as shown in Figure 8. The three apertures were arranged at 120° intervals around the optical axis. Each aperture was laterally displaced from the center such that the resulting illumination angle was approximately 1.0° with respect to the sample surface. The lateral displacement between apertures was 2.6 mm, and each aperture had a diameter of 0.8 mm. LED sources were placed behind the apertures to provide uniform illumination. Optical bandpass filters corresponding to red (No. 29), green (No. 47), and blue (No. 61) (Wratten filters, Eastman Kodak Company, Rochester, NY, USA) were used to spectrally encode each illumination direction. This configuration allows the reflected light components from different illumination directions to be separated using the RGB channels of a standard color camera.

The measurement principle is based on detecting specular reflection from each illumination direction. Incident light at a given angle is reflected toward the corresponding specular direction, producing a localized response in the associated channel. In contrast, incident light at another given angle is reflected toward the corresponding specular direction, not producing a localized response in the associated channel. This mechanism enables visualization of surface directions as a color in the normalographic image.

### 3.3. Experimental Characterization of the Telecentric Aperture

**Figure 9:**
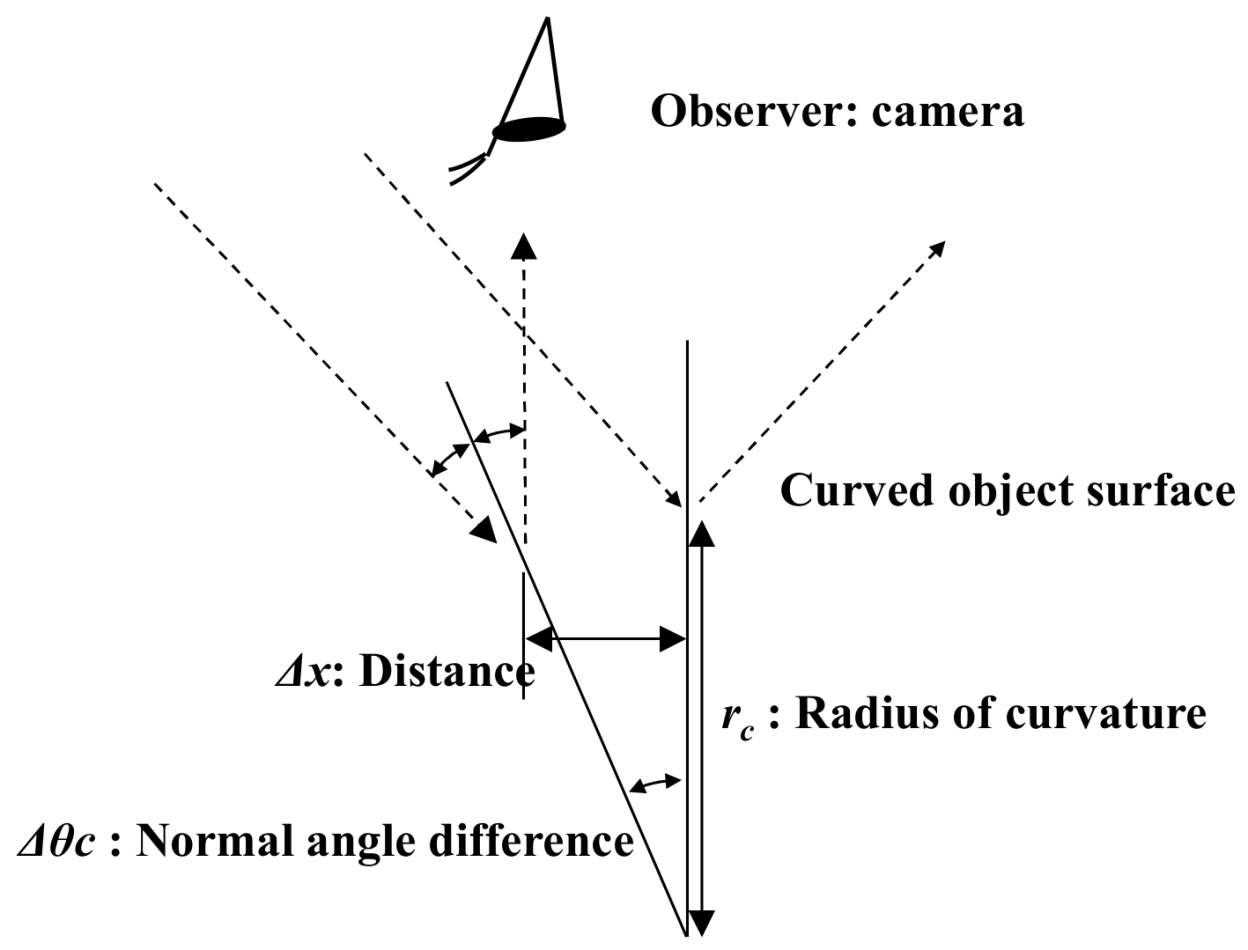
Schematic diagram of directional illumination method on a curved surface. The normal angle difference, Δ*θ*c, can be calculated from the position distance, Δ*x*, and the radius of curvature, *r_c_*.

The aperture of the telecentric optics shown in Figure 6 is adjustable. We examined the intensity corresponding to the surface normal angle. A mirror with a known radius of curvature, *r* = 580 mm, was set as a sample in Figure 9.

As shown in Figure 9, the normal angle at each position of the curved surface was obtained by calculation, as follows:

(4) $$\Delta \theta c=\arcsin\left(\Delta x/r_{c}\right),$$

**Figure 10:**
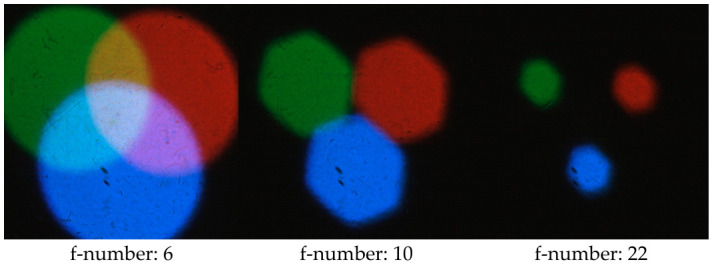
The relationship between the aperture, f-number, and the measurement angle range of a telecentric optical system. Using f-number = 6 allows for comprehensive measurement from flat surfaces to a surface normal angle of 1.1°. Therefore, an aperture of f-number = 6 is used.

Figure 10 shows the relationship between the aperture and the measurement angle range of a telecentric optical system. It can be seen that widening the aperture evenly expands the measurement angle range. Fortunately, using f-number = 6 allows for comprehensive measurement from flat surfaces to a surface normal angle of about 1.1°. Therefore, an aperture of f-number = 6 is used.

**Figure 11:**
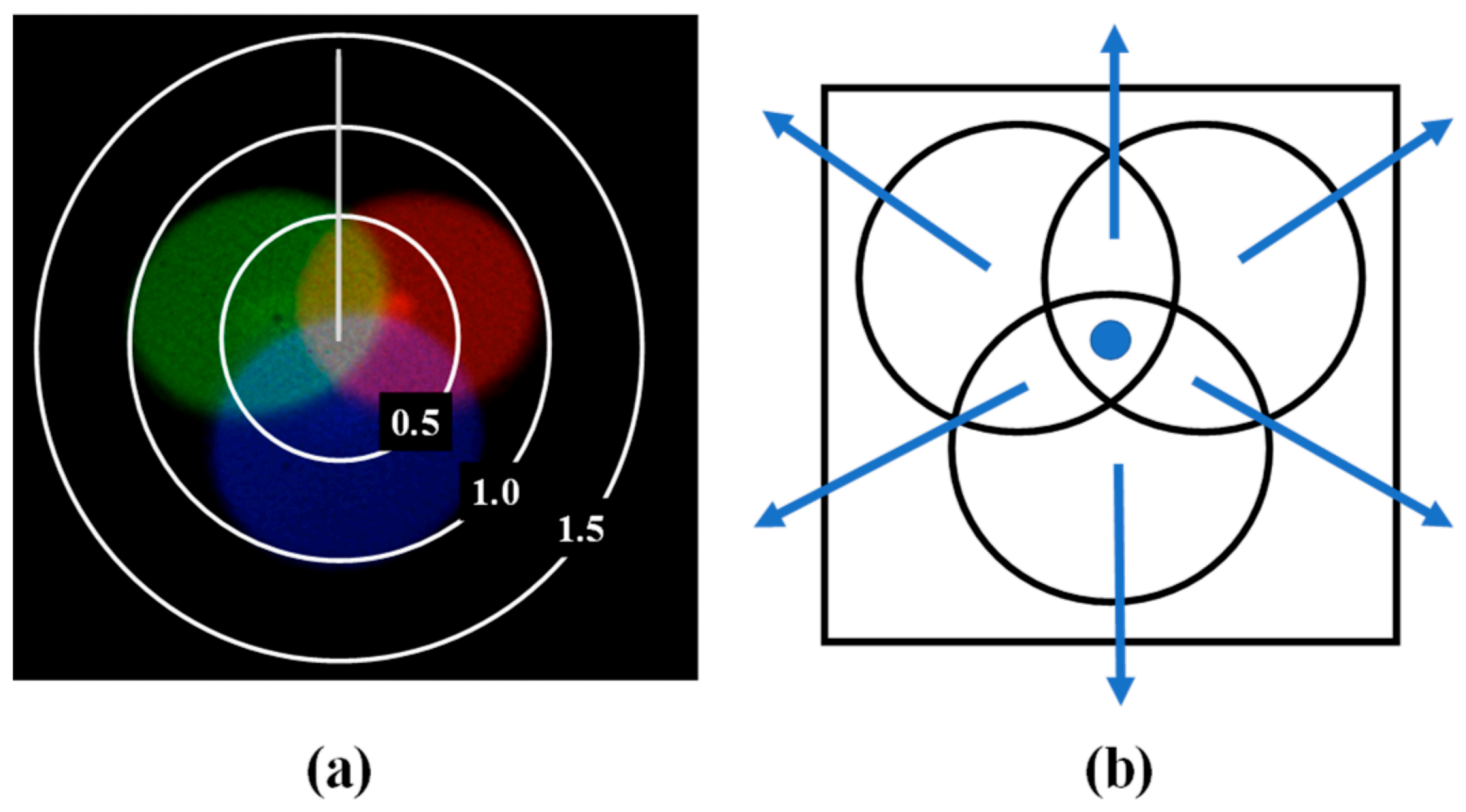
(**a**) The measurement results for a curved surface. (**b**) Schematic diagram of the eight normal directions that can be estimated from each color component.

The measurement results are shown in Figure 11a. The red, green, and blue reflected light components are distributed according to the surface normal angle. As shown in Figure 11b, angular information of at least eight facets can be obtained in terms of color components. Measurements with overlapping RGB components in the center represent flats. Six normal directions can be estimated from each color component. Regions with no reflected light and no RGB components are facets with normals greater than about 1.1 degrees. We designate this as the eighth normal-direction category. Pixels assigned to this category indicate that the local surface normal lies outside the measurable angular range. This information is useful for detecting scratches, contaminants, or other defective surface regions.

### 3.4. Measurement Results

Normalographic images were obtained for six flat samples, including an aluminum sheet, acrylic board, pottery tile, glossy-coated paper, and two types of inkjet paper.

**Figure 12:**
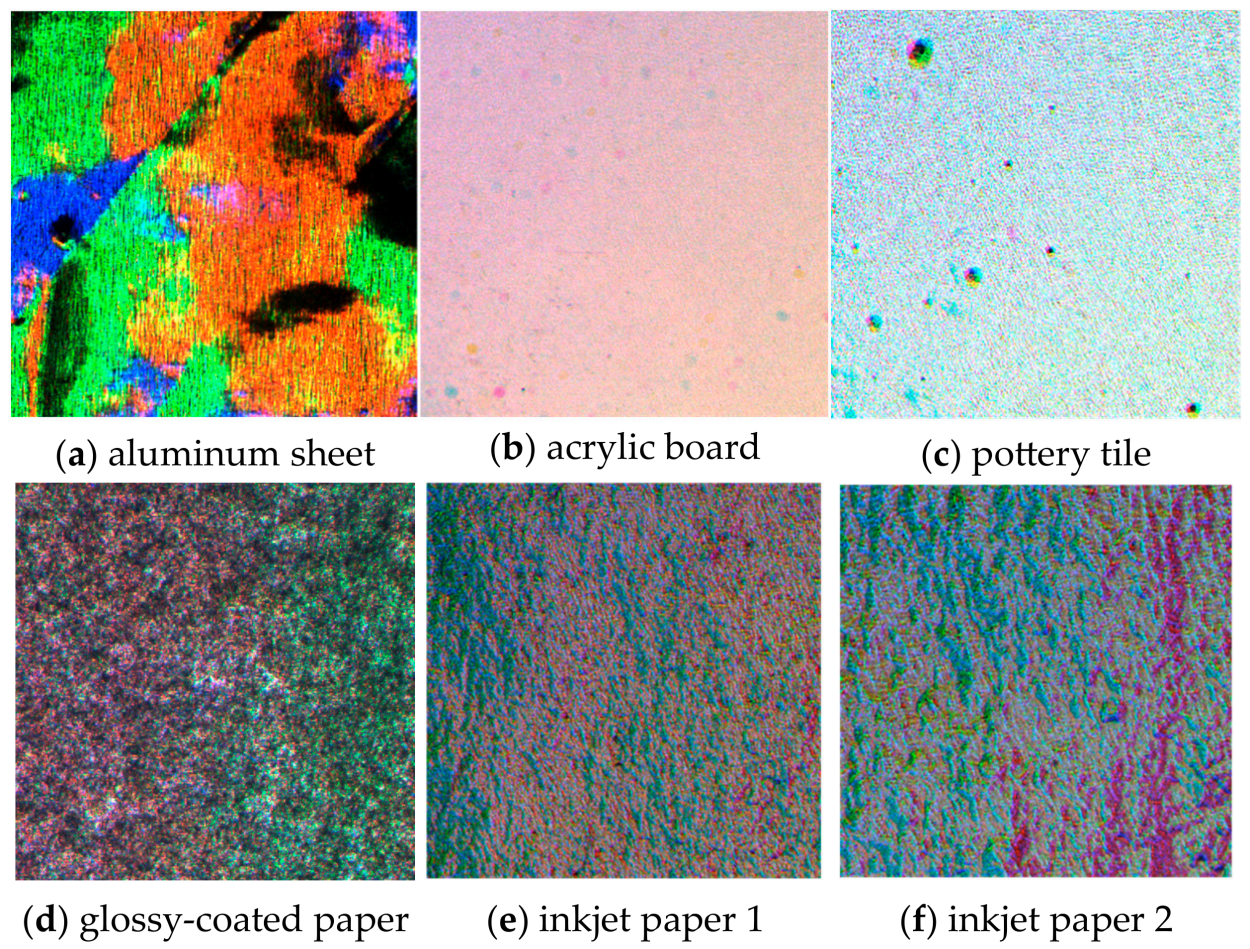
Sample results of normalography. For each material—aluminum sheet, acrylic board, pottery tile, glossy-coated paper, glossy inkjet paper 1 and glossy inkjet paper 2—the normalographic images are shown.

Figure 12 shows representative results. Although all samples are nominally flat, the normalographic images exhibit distinct spatial patterns depending on surface material. The acrylic board shows a nearly uniform response, indicating minimal variation in surface normal distribution. In contrast, glossy-coated papers and ceramic tiles exhibit structured variations in the intensity distribution, reflecting differences in surface micro-geometry and gloss characteristics. Even between two inkjet papers of similar appearance, measurable differences in normalographic patterns are observed, suggesting sensitivity to subtle surface condition variations. The aluminum sheet exhibits relatively large-scale variations in the normalographic image, corresponding to macroscopic surface undulations.

### 3.5. Verification of Measurement Accuracy

**Figure 13:**
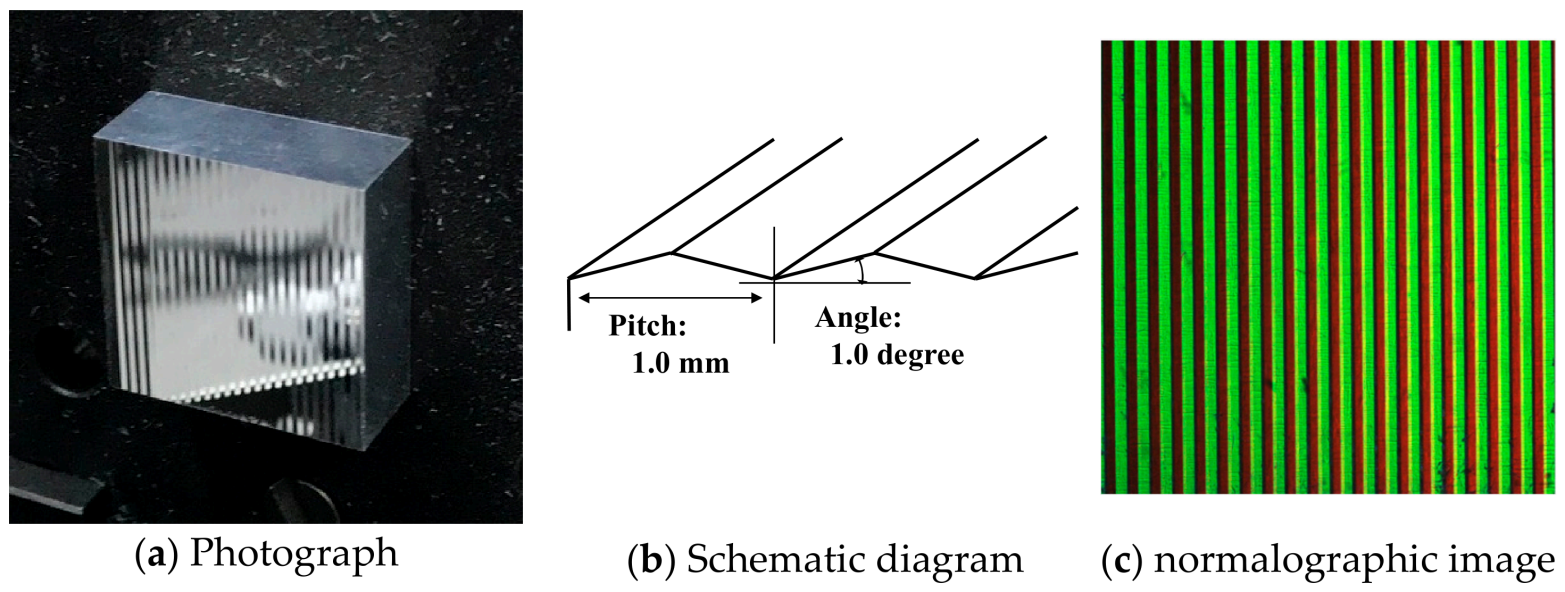
Verification of measurement accuracy: calibration sample. (**a**) Photograph of the aluminum calibration sample. (**b**) Schematic diagram of the calibration sample geometry. (**c**) Normalographic image. The measured normalographic image shows alternating responses corresponding to the designed stripe geometry.

To evaluate the measurement performance, a calibration sample with a known surface geometry was used, as shown in Figure 13. The sample consisted of an aluminum structure with a periodic stripe pattern (pitch: 1.0 mm, stripe width: 0.5 mm) and surface tilt angles of +1.0° and −1.0°. The surface provides strong specular reflection. The calibration sample was fabricated from aluminum by Toray Precision Inc. (Otsu-shi, Japan).

The measured normalographic image shows alternating responses corresponding to the designed stripe geometry. The spatial period of the measured pattern agrees with the designed pitch, confirming the validity of the proposed measurement principle.

Because the current prototype classifies surface normals into discrete angular states, the calibration focuses on verifying correct state assignment rather than continuous angular estimation.

## 4. Discussion

### 4.1. Quantification of Normalographic Images

The proposed system enables not only qualitative visualization but also quantitative interpretation of surface normal distributions.

**Figure 14:**
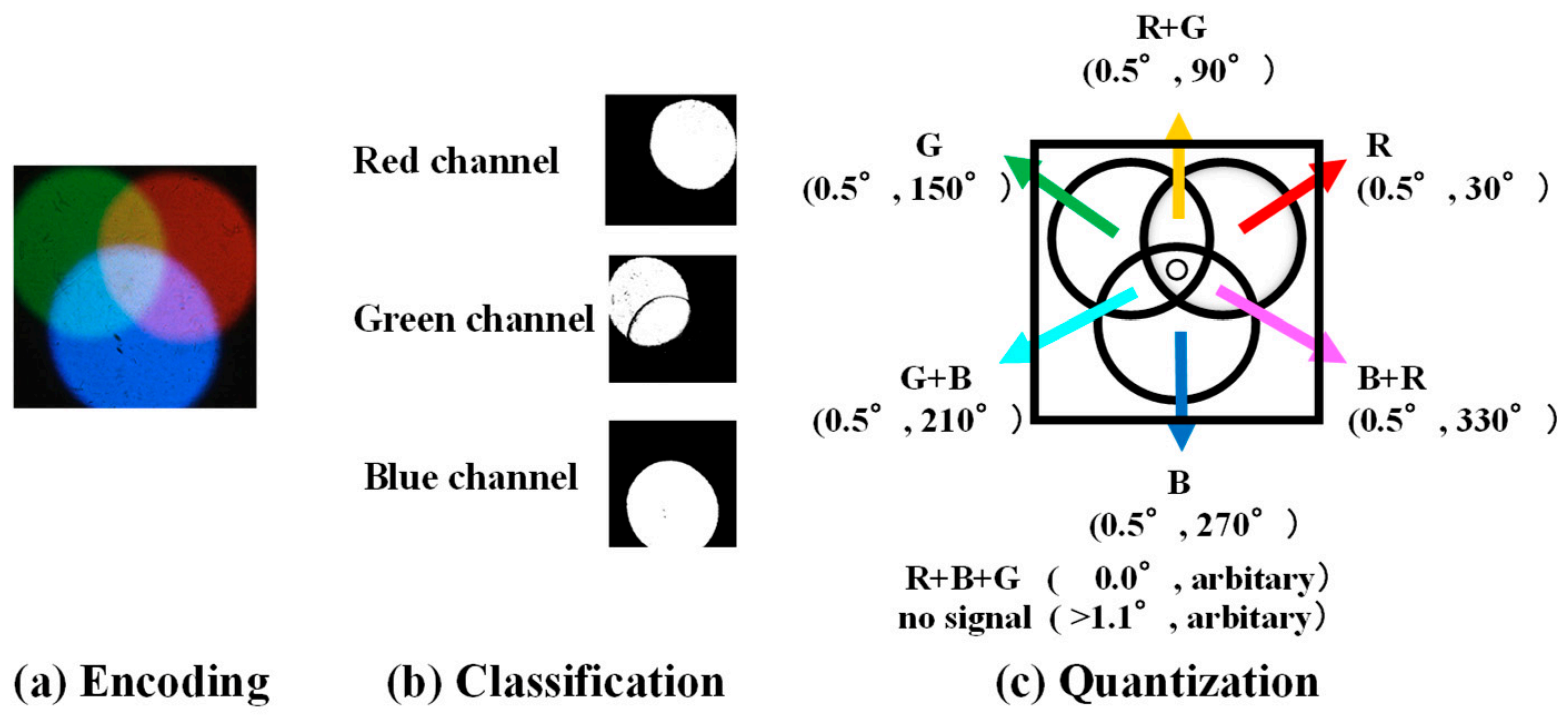
Schematic illustration of the quantization process in normalographic imaging. (**a**) Captured RGB image encoded by multi-angle illumination of a curved reference surface. (**b**) Separation of the reflected signal into R, G, and B components using the color channels of a standard camera. (**c**) Binarization of each channel enables classification of each pixel into eight discrete states corresponding to combinations of detected illumination directions (R, G, B, R+G, G+B, B+R, R+G+B, and no response). This process converts the normalographic image into a discrete surface normal representation.

Figure 14 illustrates the quantization process in normalographic imaging with a curved reference surface. Each illumination direction is encoded using a specific RGB channel. The reflected light is therefore separated in the captured image according to spectral components. Although the intensity of each channel varies due to optical and noise factors, the relative distribution of responses remains stable. Based on this property, a simple thresholding process is applied to each RGB channel to classify pixel responses. As a result, each pixel can be assigned to one of eight possible combinations of detected illumination states (R, G, B, R+G, R+B, G+B, R+G+B, and no response). These combinations correspond to discrete surface normal direction categories defined by the optical configuration.

By assigning representative angular values to each category, the normalographic image can be converted into a numerical representation of surface normal distribution. For example, the central region where all RGB channels overlap corresponds to near-flat surface normals. Regions with single-channel responses correspond to inclined surface normals associated with specific illumination directions. Regions without detectable signal correspond to surface orientations outside the measurable angular range. This binarization-based classification enables robust extraction of surface normal direction distributions despite noise and intensity fluctuations. 

The proposed system does not directly estimate continuous surface normal vectors. Instead, it encodes directional reflectance information into a discrete state space defined by the optical configuration.

### 4.2. Technical Possibilities for Performance Enhancement

The performance of the proposed system depends on the configuration of the multi-angle collimator and the spectral encoding scheme. Therefore, the system can be extended by modifying the illumination geometry.

**Figure 15:**
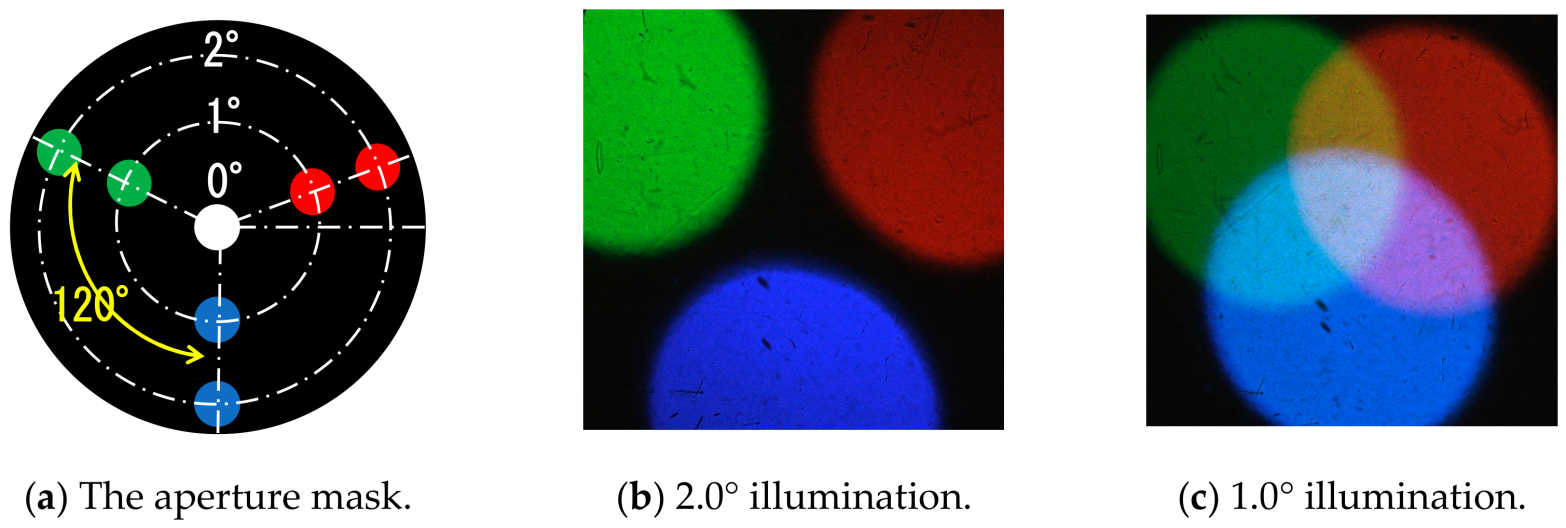
Effect of illumination geometry on normalographic imaging. (**a**) Aperture-mask configuration used to generate a 2.0° illumination angle. (**b**) Normalographic image of a curved reference surface obtained using the 2.0° illumination configuration. (**c**) Corresponding result obtained using the 1.0° illumination configuration. The comparison illustrates that the detectable range of surface normal directions can be modified through illumination design.

Figure 15 shows an example of an improved configuration in which the illumination angle is set to 2.0°, compared with the 1° configuration used in the prototype. The results demonstrate that different angular ranges of surface normals can be selectively emphasized depending on the illumination design. This indicates that the detectable angular range is not fixed but can be designed by adjusting the optical configuration. Although the current implementation uses a binarization-based classification scheme, multiple acquisitions with different illumination settings allow finer angular discrimination by combining classification results from multiple images.

In addition, the proposed normalographic representation can be further extended by increasing the number of illumination channels. While the present system uses an RGB camera, the use of a multispectral imaging system would enable an expansion of the number of independently encoded illumination directions beyond three channels. This suggests that the proposed normalography framework is scalable in terms of both angular resolution and the number of distinguishable surface normal states.

### 4.3. Applicable Surface Types and Limitations

The measurable angular range of the proposed system is approximately within ±1.1°, which constrains the types of surfaces that can be analyzed. This limitation arises from the angular range of the illumination system and the requirement that specular reflections remain within the observation range of the telecentric imaging optics. Consequently, the method is best suited for surfaces that are locally near-planar and exhibit moderate to strong specular reflectance.

It should be noted that the proposed method is not intended for general three-dimensional shape measurement. Rather, it targets industrial materials that are macroscopically flat but exhibit subtle local surface normal variations that strongly influence optical appearance. Examples include coated papers, polymer films, painted surfaces, ceramic tiles, and polished metal sheets, where local normal variations are typically confined to a few degrees.

In contrast, the method is not suitable for surfaces with large slopes or complex geometries, where the local surface normals fall outside the measurable angular range. Furthermore, for nearly ideal mirror-like surfaces, such as optical mirrors or semiconductor wafers, interferometric techniques may provide substantially higher sensitivity to extremely small surface deviations. For metallic surfaces, the influence of anisotropic reflectance and metallic luster should also be considered when interpreting the resulting normalographic images.

### 4.4. Comparison with Existing Surface Normal Measurement Techniques

Various optical methods have been proposed for measuring surface geometry and surface normal distributions. Although the proposed method shares some optical components with existing techniques, its measurement principle and intended application differ substantially.

Conventional reflectometry (gonio-reflectometry) provides highly accurate angular measurements but is inherently limited to point-wise acquisition and therefore requires mechanical scanning to obtain spatial distributions. Our previous work achieved quantitative surface normal measurement with high angular precision using sequential scanning [22]. In contrast, the present work prioritizes real-time imaging by introducing a multi-angle collimator illumination unit capable of simultaneous multi-directional illumination.

Deflectometry and phase-measuring deflectometry estimate surface shape or surface normals from distortions of projected patterns reflected by specular surfaces. These methods generally require accurate calibration, pattern projection, and computational reconstruction. The proposed approach differs fundamentally in that it directly acquires specular reflection responses under controlled illumination angles and therefore does not require pattern projection or reconstruction from distorted images.

Photometric stereo estimates surface normals from multiple images acquired under varying illumination conditions and has been developed mainly for diffuse surfaces. Although extensions to specular surfaces have been proposed using BRDF models or learning-based approaches, these methods generally depend on reflectance modeling or computational optimization. In contrast, the proposed method directly classifies surface normal states from measured specular reflections without BRDF acquisition or surface normal reconstruction.

Interferometric, confocal, and stylus-based methods first measure surface topography and subsequently derive surface normals from local height gradients. Consequently, the estimated normals are influenced by numerical differentiation and measurement noise. In contrast, the proposed method directly measures surface normal information through specular reflection rather than indirectly from surface geometry.

Consequently, the proposed method should be regarded as an appearance-oriented surface normal imaging technique rather than a general-purpose surface-metrology system. Its principal advantage lies in enabling real-time visualization of surface normal distributions through the newly developed multi-angle collimator illumination unit.

### 4.5. Industrial Robustness

A key advantage of the proposed system is its robustness for industrial implementation. During image acquisition, the apparatus contains no moving optical components or scanning mechanisms. Both the illumination unit and the observation optics remain fixed, minimizing alignment errors and reducing mechanical complexity.

The illumination unit employs collimated light generated by the proposed multi-angle collimator. Because the illumination is collimated, the incident angle is essentially independent of the distance between the sample surface and the illumination optics. Consequently, moderate displacement of the sample along the optical axis does not significantly affect the measurement geometry. The observation system employs telecentric optics, which provide both a constant observation direction across the field of view and a relatively large depth of field. This tolerance is remarkable considering that the system simultaneously achieves a spatial sampling interval of 20 μm and angular discrimination below 1°.

Since all angular information is obtained in a single exposure, the proposed method is inherently less sensitive to vibration than sequential scanning methods that require multiple images acquired over time. These characteristics enable camera-like operation in which measurements are obtained simply by placing the sample within the field of view, without optical adjustment or mechanical scanning during operation. This simplicity makes the proposed system particularly attractive for online industrial inspection. It should be emphasized that the robustness of the proposed system is achieved not by sophisticated computational compensation but by the optical design itself, namely the combination of the multi-angle collimator illumination unit and telecentric imaging optics.

## 5. Conclusions

A novel normalography technique for imaging pixel-wise surface normal distributions was proposed and demonstrated. The method combines a multi-angle collimator with multispectral illumination encoding, enabling simultaneous acquisition of directional reflectance information over an entire image without mechanical scanning. A prototype surface normal imaging camera was developed with an image size of 1024 × 1024 pixels and a spatial sampling interval of 0.02 × 0.02 mm. The current implementation classifies surface normal directions into eight discrete categories within a measurable angular range of approximately ±1.1°, allowing real-time visualization of surface normal distributions. Normalographic images were successfully obtained for a variety of industrial materials, including polymeric materials, ceramic, paper, and metallic surfaces. The results demonstrated that differences in surface condition and appearance-related characteristics can be visualized through variations in surface normal distribution. The proposed system represents not only an integration of existing optical concepts but also the development of a new illumination architecture that enables practical real-time surface normal imaging. The proposed technique is particularly suited to industrial appearance evaluation and production monitoring, where subtle variations in gloss, texture, and surface uniformity are important. Future work will focus on extending the measurable angular range and increasing the number of distinguishable surface normal states through additional illumination configurations and multispectral imaging. Furthermore, the measured surface normal distributions will be utilized for physically based reflectance rendering to reproduce visual appearance, establishing a quantitative link between surface geometry and human visual perception. We expect this framework to support industrial quality inspection, as well as appearance-oriented material design and surface engineering.

## Data Availability

The data presented in this study are available on request from the corresponding author due to privacy concerns.
